# Diagnostic Value of Serum Pepsinogen and *Helicobacter pylori* Infection in Gastric Cancer Screening in Western Zhejiang

**DOI:** 10.1155/grp/9113753

**Published:** 2026-03-01

**Authors:** Dong-Hai Yan, Yu-Fang Li

**Affiliations:** ^1^ Department of Gastroenterology, The First People′s Hospital of Jiande, Zhejiang, China; ^2^ Department of Infectious Disease, The First People′s Hospital of Jiande, Zhejiang, China

**Keywords:** diagnostic model, early gastric cancer, gastric cancer, *Hp* antibody, pepsinogen, screening

## Abstract

**Objective:**

The aim of this study is to assess the screening value of serum pepsinogen (PG) expression and *Helicobacter pylori* (*Hp*) infection for gastric cancer (GC) in western Zhejiang.

**Methods:**

A retrospective analysis was conducted on patients who underwent gastroscopy at the First People′s Hospital of Jiande between July 2020 and July 2023. Participants were classified into four groups: chronic nonatrophic gastritis, chronic atrophic gastritis, peptic ulcer, and GC, which included early gastric cancer (EGC) and advanced GC. Serum pepsinogen I (PGI), pepsinogen II (PGII), pepsinogen ratio (PGR), and anti‐*Helicobacter pylori* immunoglobulin G (Hp‐IgG) levels were measured. Group differences were assessed, and receiver operating characteristic (ROC) curve analysis was used to assess the diagnostic performance of PG alone and in combination with Hp‐IgG, age, and sex for GC and EGC.

**Results:**

Significant differences were observed among the four groups in PGI, PGII, PGR, *Hp* infection rate, age, and sex (*p* < 0.01). In benign gastric diseases, PGI and PGII levels increased with the severity and activity of gastric mucosal inflammation (*p* < 0.05). PGII levels were associated with tumor size and Lauren classification (*p* < 0.05), while PGR was associated with GC stage (*p* = 0.021). The area under the ROC curve (AUC) for PG alone in differentiating GC/EGC from benign gastric diseases ranged from 0.598 to 0.813, whereas the model incorporating PG, Hp‐IgG, age, and sex achieved an AUC of 0.851.

**Conclusion:**

Serum PG expression and *Hp* infection rates differed between patients with GC and those with benign gastric diseases in western Zhejiang. Models combining PG with demographic variables demonstrated a good diagnostic value for GC, including EGC, supporting their potential application in noninvasive GC screening.

## 1. Introduction

Over the past four decades, gastric cancer (GC) has remained the fifth most prevalent malignant tumor and the third leading cause of cancer‐related mortality worldwide [1]. In China, approximately 679,000 new cases of GC are diagnosed annually, with about 498,000 GC‐related deaths, accounting for nearly half of the global total [2]. A substantial proportion of patients diagnosed with GC are identified at an advanced stage, which is associated with a poor prognosis and a 5‐year survival rate of only 20%–30%. In contrast, the 5‐year survival rate for early gastric cancer (EGC) can reach 90% [3]. EGC can be managed through endoscopic mucosal resection or endoscopic submucosal dissection (ESD), which are minimally invasive techniques providing therapeutic outcomes comparable to surgical intervention [4]. These procedures preserve the gastric structure, thereby supporting better postoperative quality of life. Consequently, early detection and intervention for GC are of considerable clinical importance.

GC, particularly EGC, often presents without specific symptoms [5]. Although endoscopy combined with biopsy remains the gold standard for GC diagnosis, its application in large‐scale screening is limited by invasiveness, patient discomfort, lack of cost‐effectiveness, and other practical constraints [6]. Pepsinogen (PG), a precursor of pepsin, is classified into pepsinogen I (PGI) and pepsinogen II (PGII) based on biochemical properties and immunogenicity, with each type produced by different gastric mucosal cells [7]. PG levels reflect the functional status of the gastric mucosa, and approximately 1% of PG enters the bloodstream. Measurement of serum PG levels has been proposed as a noninvasive method for identifying populations at a high risk of GC, including EGC [8–14].

The serum PG level is influenced by multiple factors, including geographic region, ethnicity, age, sex, and the degree of differentiation and progression of GC, which contributes to variability in its diagnostic performance for GC [7, 15, 16]. The aim of this study was to assess serum PG expression levels and *Helicobacter pylori* (*Hp*) immunoglobulin G (Hp‐IgG) antibodies in patients with gastric diseases in western Zhejiang and to assess the diagnostic value of PG alone and in combination with other variables for GC, particularly EGC. The findings are intended to support the development of a simple and effective noninvasive screening approach for the early detection of GC in this region.

## 2. Data and Methods

### 2.1. Participants

Patients who underwent gastroscopy and biopsy, excluding those with duodenal ulcer, at the First People′s Hospital of Jiande between July 2020 and July 2023 were enrolled. Exclusion criteria were applied after medical histories were reviewed and included (1) a history of gastric surgery, (2) use of proton pump inhibitors or other gastric medications within the preceding month, (3) a history of other malignant tumors, and (4) a prior diagnosis of GC with receipt of antitumor treatment. Patients meeting the inclusion criteria provided written informed consent, and 5 mL of peripheral blood was collected for the detection of PG and anti‐Hp‐IgG.

A total of 497 patients were included in the study, with a mean age of 64.1 ± 13.1 years. The cohort comprised 317 males (63.8%) and 180 females (36.2%), and 286 patients (57.5%) tested positive for *Hp*. From the gastroscopy and biopsy findings, patients were categorized into the chronic nonatrophic gastritis (CNAG) group (*n* = 148, 29.8%), the chronic atrophic gastritis (CAG) group (*n* = 36, 7.2%), the peptic ulcer (PU) group (*n* = 41, 8.2%), and the GC group (*n* = 272, 54.7%). Among the GC group, 120 patients underwent surgical resection or ESD and had complete pathological data. Of these, 41 patients (34.2%) had EGC and 79 patients (65.8%) had advanced gastric cancer (AGC). According to the Lauren classification, 65 patients (54.1%) had intestinal‐type GC and 55 patients (45.9%) had diffuse‐type GC.

The protocol was reviewed and approved by the Ethics Committee of the First People′s Hospital of Jiande, and all procedures and examinations were conducted with a written informed consent of the patients.

### 2.2. Serum PG and *Hp* Antibody Detection

Serum was separated from peripheral venous blood samples and stored at −80°C until testing. Serum concentrations of Hp‐IgG, PGI, and PGII were measured using the enzyme‐linked immunosorbent assay (ELISA), and the pepsinogen ratio (PGR) was subsequently calculated. ELISA kits for both assays were obtained from Biohit (Finland), and all experimental procedures were performed in strict accordance with the instructions provided by the manufacturer.

### 2.3. Statistical Processing

The distribution characteristics of measurement indexes were assessed using the Kolmogorov–Smirnov test. Normally distributed measurement indexes were expressed as mean ± standard deviation ($\overset{¯}{x}\pm S$) and compared using the *t*‐test or one‐way ANOVA. Nonnormally distributed measurement indexes were expressed as quartiles (M(Q1–Q3)) and compared using the Mann–Whitney *U* test or the Kruskal–Wallis test. Categorical variables were expressed as frequency and percentage, with comparisons performed using the chi‐squared test or Fisher′s exact probability test. A *p* value < 0.05 was considered indicative of statistical significance. Receiver operating characteristic (ROC) curves and the area under the curve (AUC) were used to assess the diagnostic value of individual indexes and diagnostic models. Diagnostic models were established using multivariate binary logistic stepwise regression analysis. All statistical analyses were performed using SPSS 22.0 software (IBM Corp., Armonk, New York, United States).

## 3. Results

### 3.1. Serum PG Levels and Hp‐IgG Positivity Rates Across Groups

Sex, age, Hp‐IgG, PGI, PGII, and PGR levels were statistically analyzed for patients across the four groups. Significant differences were observed in all indices among the groups. The GC group demonstrated the highest mean age, the greatest proportion of males, the highest median PGI and PGII levels, and the lowest PGR. The PU group exhibited the highest Hp‐IgG positivity rate. No significant differences were identified between the CNAG and CAG groups for any indices except age (Table 1). Notably, while the median PGR showed a decreasing trend from the CNAG group (7.8) to the CAG group (7.0), this difference did not reach statistical significance (*p* > 0.05, Table 1). The lack of significance may be attributed to the relatively small sample size of the CAG group (*n* = 36) compared to the CNAG group (*n* = 148), and the potential influence of confounding variables such as age, sex, and *Hp* infection status on PGR levels.

**Table 1:** Comparison of demographic characteristics, *Hp* antibody status, and pepsinogen levels among patient groups.

	Group [*n* (%), $\overset{¯}{x}\pm S$ or median (M(Q1–Q3)]					*p*
	CNAG (*n* = 148)	CAG (*n* = 36)	PU (*n* = 41)	GC (*n* = 272)	*F*	
Sex					22.111^a^	< 0.001
Male	79 (53.4)	15 (41.7)	29 (70.7)^&^	194 (71.3) ^∗∗^^&&^		
Female	69 (46.6)	21 (58.3)	12 (29.3)^&^	78 (28.7) ^∗∗^^&&^		
Age (year)	55.5 ± 11.6	63.1 ± 12.4^∗∗^	61.7 ± 13.5^∗^	69.4 ± 11.2^∗∗&&##^	46.544^b^	< 0.001
Hp					17.172^a^	0.001
Positive	83 (56.1)	21 (58.3)	36 (87.8) ^∗∗^^&^	146 (53.7)^##^		
Negative	65 (43.9)	15 (41.7)	5 (12.2) ^∗∗^^&^	126 (46.3)^##^		
PGI (*μ*g/L)	68.0 (49.0–106.0)	68.0 (49.5–103.2)	106.0 (80.0–177.5) ^∗^	117.0 (60.3–192.0) ^∗∗^	24.284^c^	< 0.001
PGII (*μ*g/L)	9.2 (5.7–15.8)	8.3 (6.5–16.6)	15.0 (10.1–33.5) ^∗∗^	28.0 (14.0–53.5) ^∗∗^^&&^	108.201^c^	< 0.001
PGR	7.8 (6.2–9.3)	7.0 (5.6–9.2)	5.9 (4.8–8.9)	3.4 (2.3–5.5) ^∗∗^^##&&^	149.532^c^	< 0.001

^a^Represents the chi‐square test.

^b^Represents the one‐way ANOVA.

^c^Represents the Kruskal–Wallis test.

^∗^ *p* < 0.05.

^∗∗^ *p* < 0.01, vs. the CNAG group.

^&^ *p* < 0.05.

^&&^ *p* < 0.01, vs. the CAG group.

^##^ *p* < 0.01, vs. the PU group.

### 3.2. Comparison of Serum PG Levels According to Pathological Feature Subgroups of Gastric Mucosal Biopsies in Benign Gastric Diseases

Serum PG expression levels were analyzed according to pathological feature subgroups of gastric mucosal biopsies in patients with benign gastric diseases (Table 2). PGI and PGII levels were found to increase with a greater degree and activity of gastric mucosal inflammation, with the differences reaching statistical significance (*p* < 0.05). Levels decreased with worsening gastric mucosal atrophy and increased with more severe gastric mucosal intestinal metaplasia; however, these differences were not statistically significant.

**Table 2:** Comparison of serum pepsinogen levels according to pathological feature subgroups of gastric mucosal biopsies in benign gastric diseases.

	*n*	Serum PG level (median M(Q1–Q3))		
		PGI (*μ*g/L)	PGII (*μ*g/L)	PGR
Degree of inflammation				
Mild	75	69.0 (49.0–107.0)	8.7 (5.7–15.4)	7.8 (6.1–9.5)
Moderate	26	94.5 (62.7–211.7)	14.9 (9.7–25.4)	6.4 (5.5–8.6)
Severe	6	153.0 (72.0–226.0)	17.6 (8.6–43.2)	6.8 (4.3–11.7)
*F*		7.664	8.143	2.377
*p*		0.022	0.017	0.305
Activity of inflammation				
Inactive	176	69.5 (49.0–111.0)	8.9 (5.7–15.8)	7.7 (4.2–9.3)
Active	45	95.0 (58.5–154.0)	14.7 (10.2–23.5)	6.1 (4.5–9.1)
*Z*		−2.578	−3.659	−2.498
*p*		0.010	< 0.001	0.013
Atrophy				
Nonatrophic	183	76.0 (51.0–124.0)	10.0 (6.0–19.3)	7.7 (5.7–9.3)
Atrophic	38	68.0 (50.5–106.2)	8.3 (6.5–16.4)	7.0 (5.5–9.1)
*Z*		−0.862	−0.258	−0.891
*p*		0.389	0.796	0.373
Degree of intestinal metaplasia				
None	142	70.0 (49.0–119.0)	9.5 (5.4–17.4)	7.9 (5.9–9.5)
Mild	69	77.0 (54.5–120.5)	10.2 (6.7–17.7)	7.1 (6.0–8.5)
Moderate–severe	10	92.5 (60.2–154.7)	15.8 (6.4–34.2)	6.2 (4.2–8.0)
*F*		1.686	3.065	4.338
*p*		0.430	0.216	0.114

*Note:* In the pathological reports of some cases, the degree of inflammation was not graded and not included in the analysis; *F* represents the Kruskal–Wallis test; *Z* represents the Mann–Whitney *U* test.

### 3.3. Comparison of Serum PG Levels Among GC Subgroups

Among the 120 patients with GC who underwent endoscopic or surgical treatment, PGI, PGII, and PGR levels were analyzed according to complete postoperative pathological feature subgroups (Table 3). PGII expression levels were associated with tumor size and Lauren classification, and PGR values were higher in EGC than in AGC (*p* = 0.021). No significant differences in PGI, PGII, or PGR levels were observed among subgroups defined by tumor site, tissue type, degree of cellular differentiation, or clinical TNM stage.

**Table 3:** Comparison of serum PG levels among GC subgroups.

	*n*	Serum PG level (median M(Q1–Q3))		
		PGI (*μ*g/L)	PGII (*μ*g/L)	PGR
Tumor site				
Gastric fundus and cardia	16	132.5 (88.8–250.0)	27.0 (16.1–77.5)	4.2 (2.3–6.8)
Gastric body	28	142.6 (74.3–224.8)	40.9 (17.5–71.5)	3.9 (2.7–5.9)
Gastric angle	21	117.0 (80.5–175.5)	32.0 (20.0–49.5)	2.8 (2.4–4.9)
Gastric antrum	55	120.0 (43.0–209.0)	24.0 (12.0–62.0)	3.4 (2.0–5.5)
*F*		2.726	3.127	1.183
*p*		0.436	0.372	0.612
Tumor size (cm)				
< 3	55	117.0 (65.5–190.3)	26.0 (12.0–46.0)	3.8 (2.8–6.5)
3~5	32	133.0 (67.5–250.0)	37.0 (24.0–89.0)	3.2 (1.7–5.3)
> 5	31	131.0 (65.6–209.0)	30.0 (16.0–72.0)	3.2 (2.0–5.4)
*F*		1.186	7.602	4.617
*p*		0.553	0.022	0.099
Histologic type				
Adenocarcinoma	88	126.5 (62.2–220.5)	28.0 (14.0–52.0)	3.7 (2.5–5.6)
Signet‐ring cell carcinoma	32	131.0 (76.6–186.3)	40.4 (18.0–73.7)	3.0 (1.9–5.6)
*Z*		−0.315	−1.600	−1.095
*p*		0.753	0.110	0.274
Lauren classification				
Intestinal type	65	122.0 (56.1–202.5)	24.0 (12.0–50.0)	3.8 (2.2–5.8)
Diffuse type	55	131.0 (78.2–226.0)	38.0 (22.0–72.0)	3.3 (2.2–5.2)
*Z*		−1.441	−2.629	−0.798
*p*		0.150	0.009	0.425
Differentiation degree				
Poorly differentiated	58	129.0 (73.1–194.0)	33.1 (17.3–57.0)	3.3 (2.1–5.1)
Poorly to moderately differentiated	30	133.5 (88.7–210.2)	26.0 (15.5–70.5)	3.6 (2.2–6.7)
Moderately differentiated	20	115.0 (61.4–243.7)	30.0 (16.5–53.0)	3.9 (2.6–6.8)
Well differentiated	12	65.5 (32.2–184.0)	16.0 (12.0–38.9)	3.3 (2.3–5.5)
*F*		2.987	2.503	1.055
*p*		0.394	0.475	0.788
cTNM stage				
I	51	120.0 (62.0–200)	22.0 (12.0–46.0)	4.0 (2.6–8.0)
II	7	77.0 (9.0–350.0)	44.0 (22.0–110.0)	1.7 (0.9–3.7)
III	13	138.0 (87.5–209.0)	38.0 (25.0–50.0)	3.4 (3.1–4.4)
IV	49	132.0 (69.3–244.0)	30.0 (15.7–73.7)	3.3 (2.0–5.5)
*F*		2.133	6.007	6.350
*p*		0.545	0.111	0.096
Pathological stage				
Early	41	126.0 (63.0–202.5)	26.0 (12.0–49.5)	4.0 (2.8–8.3)
Advanced	79	127.0 (69.0–214.0)	30.0 (18.0–70.0)	3.2 (2.0–5.2)
*Z*		−0.163	−1.943	−2.299
*p*		0.870	0.052	0.021

*Note:* *F* represents the Kruskal–Wallis test; *Z* represents the Mann–Whitney *U* test. Early gastric cancer was defined according to the Chinese consensus guidelines for gastric cancer diagnosis and treatment as tumor invasion confined to the mucosa and submucosa, irrespective of lesion size or lymph node metastasis.

### 3.4. Diagnostic Value of Serum PG as a Single Index and in Combination With Multiple Indexes for GC and EGC

The ROC curve was applied to assess the diagnostic value of PGI, PGII, and PGR individually and in combination with multiple indices for differentiating GC and EGC from benign gastric diseases (Figure 1). For the combined analysis, GC was designated as the case group and benign gastric diseases (CNAG + CAG + PU) as the control group. Three diagnostic models were developed using binary logistic stepwise regression analysis: (1) a model based on the combination of PGI, PGII, and PGR; (2) a model combining PG (PGI, PGII, and PGR) with *Hp*; and (3) a model combining PG (PGI, PGII, and PGR) with *Hp*, age, and sex:

Figure 1(a, b) ROC curves of serum PG as a single index and in combination with other indices for differentiating GC from benign gastric diseases.

**Figure:**
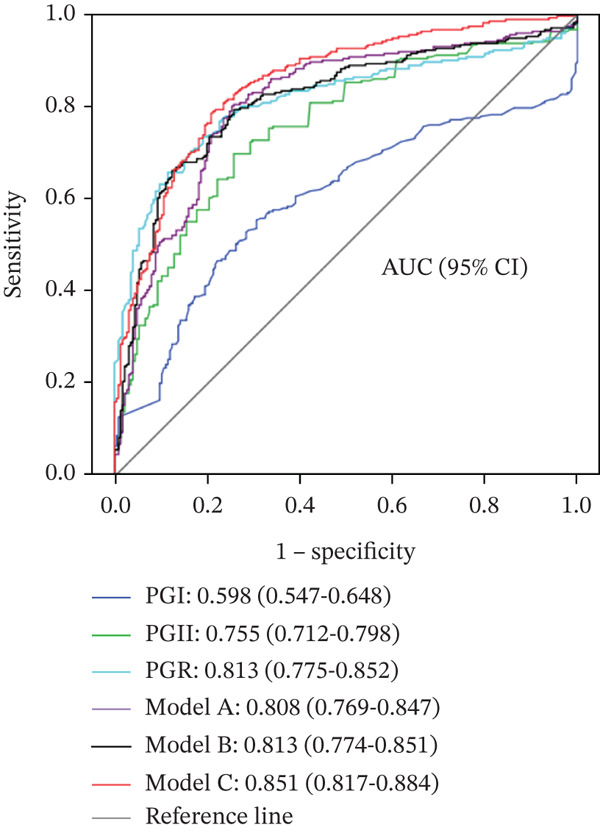
(a)

**Figure:**
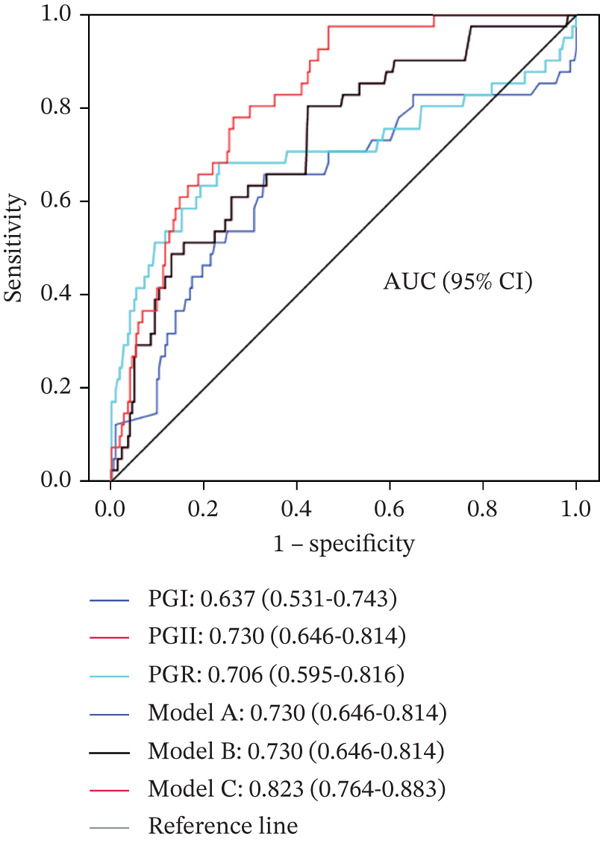
(b)

Model A (Model A): logit (p) = 0.032PGII − 0.138PGR + 0.242,

Model B (Model B): logit (p) = 0.035PGII − 0.139PGR − 0.667Hp + 0.573, and

Model C (Model C) = 0.034PGII − 0.087PGR − 1.195Hp + 0.079Age − 0.741Sex − 4.197,

where the *Hp*‐negative value is 0, the *Hp*‐positive value is 1; the value of sex = male is 0, and the value of sex = female is 1.

The variables of each model and their data are presented in Table 4.

**Table 4:** Variables and parameters of three diagnostic models based on serum PG.

Variable	*B*	SE	Wald	*p*	OR (95% CI)
Model A					
PGII	0.032	0.006	29.100	< 0.001	1.032 (1.020–1.044)
PGR	−0.138	0.033	17.027	< 0.001	0.871 (0.816–0.930)
Constant	0.242	0.290	0.697	0.404	1.274
Model B					
PGII	0.035	0.006	31.346	< 0.001	1.036 (1.023–1.048)
PGR	−0.139	0.034	16.854	< 0.001	0.871 (0.815–0.930)
Hp	−0.677	0.212	10.227	0.001	0.508 (0.336–0.769)
Constant	0.573	0.313	3.345	0.067	1.774
Model C					
PGII	0.034	0.006	29.950	< 0.001	1.035 (1.022–1.047)
PGR	−0.087	0.032	7.570	0.006	0.917 (0.862–0.975)
Hp	−1.195	0.253	22.228	< 0.001	0.303 (0.184–0.497)
Age	0.079	0.010	58.453	< 0.001	1.082 (1.061–1.105)
Sex	−0.741	0.242	9.379	0.002	0.477 (0.297–0.766)
Constant	−4.197	0.725	33.515	< 0.001	0.015

*Note:* The ROC curves of PGI, PGII, and PGR as individual indices and of the three diagnostic models for differentiating GC/EGC from benign gastric diseases are presented in Figure 1. PGI, PGII, and PGR individually demonstrated diagnostic value, with AUC values ranging from 0.598 to 0.813. Models combining PG with multiple indices further improved diagnostic performance, with AUC values ranging from 0.730 to 0.851.

A: PG as a single index and diagnostic models combining PG with multiple indices for differentiating GC from benign gastric diseases; B: PG as a single index and diagnostic models combining PG with multiple indexes for differentiating EGC from benign gastric diseases. PGI: PGI; PGII: PGII; PGR: PG ratio; *Hp*: *H. pylori*. Model A : logit (*p*) = 0.032PGII − 0.138PGR + 0.242; Model B : logit (*p*) = 0.035PGII − 0.139PGR − 0.667*H* *p* + 0.573; Model C : logit (*p*) = 0.034PGII − 0.087PGR − 1.195*H* *p* + 0.079Age − 0.741Sex − 4.197.

The diagnostic performance indices of the three PG‐based models for distinguishing GC/EGC from benign gastric diseases were calculated (Table 5). Model C demonstrated the highest diagnostic efficacy for both GC and EGC, with accuracies of 79.3% and 73.7%, respectively.

**Table 5:** Diagnostic performance indices of different pepsinogen‐based models for GC.

	Sensitivity (%)	Specificity (%)	Accuracy (%)	Positive predictive value (%)	Negative predictive value (%)	Positive likelihood ratio	Negative likelihood ratio
All GCs							
Model A	77.6	76.4	77.0	79.9	73.8	3.29	0.29
Model B	79.1	73.8	76.7	77.9	75.1	3.00	0.30
Model C	80.9	76.4	79.3	80.6	76.8	3.43	0.26
EGC							
Model A	80.5	57.7	61.2	25.7	61.9	1.90	0.33
Model B	80.5	57.7	61.2	25.7	61.9	1.90	0.33
Model C	78.0	72.9	73.7	34.4	94.8	2.88	0.30

*Note:* Model A : logit (*p*) = 0.032PGII − 0.138PGR + 0.242; Model B : logit (*p*) = 0.035PGII − 0.139PGR − 0.667Hp + 0.573; Model C : logit (*p*) = 0.034PGII − 0.087PGR − 1.195Hp + 0.079Age − 0.741Sex − 4.197.

## 4. Discussion

In this study, serum PG expression levels and Hp‐IgG positivity rates were assessed in patients with GC and benign gastric diseases in western Zhejiang. Patients with GC exhibited higher serum PGI and PGII levels and lower PGR values compared to those with benign gastric diseases. No significant difference in Hp‐IgG positivity was observed between patients with GC and those with gastritis; however, the positivity rate was lower than that observed in patients with PU. Further analysis indicated that serum PG levels were positively correlated with the degree and activity of chronic gastritis, while no significant associations were identified with pathological features or tumor stage of GC. PGI, PGII, and PGR individually demonstrated the diagnostic value for GC, including EGC, with PGII performing better than PGI and PGR showing the highest diagnostic value. The combination of the three PG indexes enhanced diagnostic performance for GC. No substantial improvement was observed when *Hp* was added to the three PG indices. However, combining PGI, PGII, and PGR with age and sex further improved the diagnostic value, yielding AUCs above 0.8 and accuracy rates of 79.3% and 73.7% for GC and EGC, respectively.

The three PG indices, whether individually or in combination, demonstrated a moderate diagnostic value for GC. Diagnostic performance was notably improved when combined with Hp‐IgG, age, and sex (Model C). Model C achieved a sensitivity of approximately 80% and a specificity exceeding 70% for all GC cases; specificity remained at 60% when sensitivity was adjusted to 90%, while sensitivity remained at 57% when specificity was adjusted to 90% (data not presented in the results). These findings indicate that Model C holds a considerable clinical value in GC screening. Owing to its simplicity and feasibility in clinical practice, Model C may serve as an effective screening tool for patients unable to undergo gastroscopy and may also be valuable for identifying high‐risk patients prior to gastroscopy.

The close association between *Hp* infection and the development of GC is well established. Some studies have reported that GC occurred exclusively in *Hp*‐positive patients, with no cases observed in *Hp*‐negative patients during follow‐up [17]. In this study, the overall *Hp* infection rate among patients with gastric diseases in western Zhejiang was 57.5%, which was generally consistent with the national average in China [18]. Compared with Model A, which incorporated the three PG indices, Model B, which combined the three PG indices with *Hp*, did not indicate a significant improvement in detection efficacy for GC or EGC. This finding indicates that the *Hp* status alone may have a limited screening value for GC in regions with high *Hp* infection prevalence, which is consistent with several previous reports [19–21].

The results of this study indicated that PGI and PGII levels increased, while PGR decreased, with increasing activity of gastric mucosal inflammation in patients with benign gastric diseases, and these differences were statistically significant. PGI and PGII levels demonstrated a positive correlation with the degree of gastric mucosal inflammation and a negative correlation with PGR, aligning with the findings of Kang et al. [22] These observations indicate that inflammation can promote PG secretion, particularly following *Hp* infection. In patients with atrophic gastric mucosa, PGI, PGII, and PGR levels were lower than those in patients with nonatrophic gastric mucosa; however, these differences were not statistically significant, which may be attributed to the small sample size and mild atrophy observed in the CAG group in this study. Notably, our subanalysis of CAG and CNAG groups revealed no statistical difference in PGR, despite a trend of lower PGR in the CAG group (7.0 vs. 7.8 in CNAG), which is consistent with the biological expectation that gastric mucosal atrophy reduces PGR by impairing PGI secretion (from gastric chief cells) while preserving PGII secretion (from mucous neck and pyloric glands). The lack of statistical significance may be further explained by two key factors: first, the substantial imbalance in sample sizes between the two groups (CNAG: *n* = 148 vs. CAG: *n* = 36), which may have limited statistical power to detect subtle differences; second, confounding variables including age, *Hp* infection status, and inflammatory activity, which are known to modulate serum PG levels and may have masked the association between atrophy and PGR. Future studies with larger cohorts of CAG patients and stratified analysis by atrophy severity, *Hp* infection status, and inflammatory activity are warranted to clarify this relationship in the western Zhejiang population. Due to the distinct distributions of cells responsible for PGI and PGII secretion, nonatrophic or mildly atrophic gastric mucosa can exhibit increased PG secretion (predominantly PGI) in response to inflammatory stimulation. With progression to severe atrophy, gastric chief cells and mucous neck cells are replaced by pyloric glands, resulting in decreased PGI secretion, while PGII levels remain largely unchanged. Consequently, the PGR declines significantly. Additionally, patients with diffuse‐type GC exhibited higher PGII levels compared to those with intestinal‐type GC, which is consistent with the findings of Ito et al., and may be related to the differing pathogenic mechanisms underlying these two GC subtypes [23].

This study has certain limitations. First, the study population comprised patients with gastric diseases in western Zhejiang; therefore, further research is required to determine whether the conclusions are applicable to patients with gastric diseases in other regions of China. Second, the small sample size of the CAG group may have affected the reliability of the results. Third, the uneven distribution of pathological specimens (primarily antral mucosa with few gastric body samples) and the substantially smaller CAG group size (*n* = 36 vs. CNAG *n* = 148) may have limited our ability to detect expected differences in PGR between atrophic and nonatrophic gastritis, as reported in the literature. Additionally, this study did not explore the relationship between PGR and OLGA/OLGIM staging, as this association is well‐documented in the literature and our focus was on developing a simplified screening model for resource‐limited settings. Future studies addressing these limitations are warranted.

## 5. Conclusion

GC develops based on gastric mucosal inflammation. To reduce the potential influence of race, dietary habits, and lifestyle, this study examined a noninvasive and straightforward screening method for GC, particularly EGC, in the population of western Zhejiang. *Hp* has been classified by international authorities as a class I carcinogen for GC, with its carcinogenic mechanism linked to the induction and progression of gastric mucosal inflammation. Given the differing distributions of PGI and PGII in gastric mucosal cells, notable differences in PG expression levels may occur under the influence of gastric mucosal inflammation. In this study, anti‐Hp‐IgG and serum PGI and PGII levels were measured using ELISA, and PGR was calculated. From the gastroscopy and biopsy pathology, patients were categorized into the CNAG, CAG, PU, and GC groups. Postoperative pathological classification of GC cases was further divided into EGC and AGC. Comparisons of serum PGI, PGII, and PGR levels, and *Hp* infection rates among the four groups were performed to further analyze the association between PG levels and gastric mucosal pathological features. The AUC of the ROC curve was applied to assess the diagnostic value of PG, both individually and in combination with multiple indices, in differentiating GC and EGC from benign gastric diseases.

From this study, a noninvasive and simple screening approach for GC in the local population was identified. However, further refinement is required due to study limitations. Incorporation of additional gastric function markers, such as gastrin levels, may further enhance screening efficacy for GC, particularly EGC.

**Nomenclature**
PG: pepsinogen
*Hp*
: *H. pylori*
GC: gastric cancer
EGC: early gastric cancer
AGC: advanced gastric cancer
ELISA: enzyme‐linked immunosorbent assay
Hp‐IgG: anti‐*Helicobacter pylori* immunoglobulin G
PGI: pepsinogen I
PGII: pepsinogen II
PGR: pepsinogen ratio
AUC: area under the curve
ROC: receiver operating characteristic curve
EMR: endoscopic mucosal resection
ESD: endoscopic submucosal dissection

## Author Contributions

Conception and design of the research, statistical analysis, obtaining financing, writing of the manuscript, and critical revision of the manuscript for intellectual content: Dong‐Hai Yan. Acquisition of data: Yu‐Fang Li. Analysis and interpretation of the data: Dong‐Hai Yan and Yu‐Fang Li.

## Funding

This work was supported by the Hangzhou Science and Technology Plan Guidance Project (Agriculture and Social Development), 2016351Y162.

## Disclosure

All authors read and approved the final draft.

## Ethics Statement

This study was conducted with approval from the Ethics Committee of The First People′s Hospital of Jiande. This study was conducted in accordance with the Declaration of Helsinki. Written informed consent was obtained from all participants.

## Consent

The authors have nothing to report.

## Conflicts of Interest

The authors declare no conflicts of interest.

## Data Availability

The datasets used or analyzed during the current study are available from the corresponding author on reasonable request.

## References

[1] Bray F. , Ferlay J. , Soerjomataram I. , Siegel R. L. , Torre L. A. , and Jemal A. , Global Cancer Statistics 2018: GLOBOCAN Estimates of Incidence and Mortality Worldwide for 36 Cancers in 185 Countries, Ca: A Cancer Journal for Clinicians. (2018) 68, no. 6, 394–424, 10.3322/caac.21492, 2-s2.0-85053395052. 30207593

[2] Chen W. , Zheng R. , Baade P. D. , Zhang S. , Zeng H. , Bray F. , Jemal A. , Yu X. Q. , and He J. , Cancer Statistics in China, 2015, CA: A Cancer Journal for Clinicians. (2016) 66, no. 2, 115–132, 10.3322/caac.21338, 2-s2.0-84960796740, 26808342. 26808342

[3] Lu J. , Huang C. M. , Zheng C. H. , Li P. , Xie J. W. , Wang J. B. , and Lin J. X. , Consideration of Tumor Size Improves the Accuracy of TNM Predictions in Patients With Gastric Cancer After Curative Gastrectomy, Surgical Oncology. (2013) 22, no. 3, 167–171, 10.1016/j.suronc.2013.05.002, 2-s2.0-84883787621, 23787074. 23787074

[4] Gotoda T. , Endoscopic Resection of Early Gastric Cancer, Gastric Cancer. (2007) 10, no. 1, 1–11, 17334711, 10.1007/s10120-006-0408-1, 2-s2.0-33847669835. 17334711

[5] Tsukuma H. , Oshima A. , Narahara H. , and Morii T. , Natural History of Early Gastric Cancer: A Non-concurrent, Long Term, Follow Up Study, Gut. (2000) 47, no. 5, 618–621, 10.1136/gut.47.5.618, 2-s2.0-0033756371, 11034575. 11034575 PMC1728114

[6] Zou W. B. , Yang F. , and Li Z. S. , How to Improve the Diagnosis Rate of Early Gastric Cancer in China, Journal of Zhejiang University. Medical Sciences. (2015) 44, no. 1, 9–14. 25851969 10.3785/j.issn.1008-9292.2015.01.002 PMC10396879

[7] Lee S. Y. , Endoscopic Gastritis, Serum Pepsinogen Assay, and Helicobacter pylori Infection, Korean Journal of Internal Medicine. (2016) 31, no. 5, 835–844, 10.3904/kjim.2016.166, 2-s2.0-84986903255, 27604795. 27604795 PMC5016293

[8] Miki K. , Ichinose M. , Ishikawa K. B. , Yahagi N. , Matsushima M. , Kakei N. , Tsukada S. , Kido M. , Ishihama S. , Shimizu Y. , Suzuki T. , and Kurokawa K. , Clinical Application of Serum Pepsinogen I and II Levels for Mass Screening to Detect Gastric Cancer, Japanese Journal of Cancer Research. (1993) 84, no. 10, 1086–1090, 10.1111/j.1349-7006.1993.tb02805.x, 2-s2.0-0027371774, 8226283. 8226283 PMC5919064

[9] Kodoi A. , Yoshihara M. , Sumii K. , Haruma K. , and Kajiyama G. , Serum Pepsinogen in Screening for Gastric Cancer, Journal of Gastroenterology. (1995) 30, no. 4, 452–460, 10.1007/BF02347560, 2-s2.0-0029095227, 7550854. 7550854

[10] Hattori Y. , Tashiro H. , Kawamoto T. , and Kodama Y. , Sensitivity and Specificity of Mass Screening for Gastric Cancer Using the Measurment of Serum Pepsinogens, Japanese Journal of Cancer Research. (1995) 86, no. 12, 1210–1215, 10.1111/j.1349-7006.1995.tb03317.x, 2-s2.0-0029417265, 8636012. 8636012 PMC5920667

[11] Kitahara F. , Kobayashi K. , Sato T. , Kojima Y. , Araki T. , and Fujino M. A. , Accuracy of Screening for Gastric Cancer Using Serum Pepsinogen Concentrations, Gut. (1999) 44, no. 5, 693–697, 10.1136/gut.44.5.693, 2-s2.0-0033042057, 10205207. 10205207 PMC1727514

[12] Yoshihara M. , Sumii K. , Haruma K. , Kiyohira K. , Hattori N. , Tanaka S. , Kajiyama G. , and Shigenobu T. , The Usefulness of Gastric Mass Screening Using Serum Pepsinogen Levels Compared With Photofluorography, Hiroshima Journal of Medical Sciences. (1997) 46, no. 2, 81–86, 9232936. 9232936

[13] Miki K. , Morita M. , Sasajima M. , Hoshina R. , Kanda E. , and Urita Y. , Usefulness of Gastric Cancer Screening Using the Serum Pepsinogen Test Method, American Journal of Gastroenterology. (2003) 98, no. 4, 735–739, 10.1111/j.1572-0241.2003.07410.x, 2-s2.0-0038292980, 12738449. 12738449

[14] Samloff I. M. , Pepsinogens, Pepsins, and Pepsin Inhibitors, Gastroenterology. (1971) 60, no. 4, 586–604, 10.1016/S0016-5085(71)80065-3, 2-s2.0-0015044485, 4324336. 4324336

[15] Bornschein J. , Selgrad M. , Wex T. , Kuester D. , and Malfertheiner P. , Serological Assessment of Gastric Mucosal Atrophy in Gastric Cancer, BMC Gastroenterology. (2012) 12, no. 1, 10.1186/1471-230X-12-10, 2-s2.0-84856324043. PMC3280182 22289789

[16] Lauren P. , The Two Histological Main Types of Gastric Carcinoma: Diffuse and So-Called Intestinal-Type Carcinoma. An Attempt at a Histo-Clinical Classification, Acta Pathologica et Microbiologica Scandinavica. (1965) 64, no. 1, 31–49, 10.1111/apm.1965.64.1.31, 2-s2.0-12644292106, 14320675. 14320675

[17] Uemura N. , Okamoto S. , Yamamoto S. , Matsumura N. , Yamaguchi S. , Yamakido M. , Taniyama K. , Sasaki N. , and Schlemper R. J. , Helicobacter pylori Infection and the Development of Gastric Cancer, New England Journal of Medicine. (2001) 345, no. 11, 784–789, 10.1056/NEJMoa001999, 2-s2.0-0035856021, 11556297. 11556297

[18] Cao X. Y. , Jia Z. F. , Jin M. S. , Cao D. H. , Kong F. , Suo J. , and Jiang J. , Serum Pepsinogen II Is a Better Diagnostic Marker in Gastric Cancer, World Journal of Gastroenterology. (2012) 18, no. 48, 7357–7361. 23326145 10.3748/wjg.v18.i48.7357 PMC3544042

[19] Li M. Y. , Zhang D. Q. , Lu X. , and Chen W. C. , Comparison of Two Serological Methods in Screening Gastric Cancer and Its Precancerous Condition, Zhonghua nei ke za zhi. (2018) 57, no. 12, 907–911, 10.3760/cma.j.issn.0578-1426.2018.12.006, 30486559. 30486559

[20] Ni D. Q. , Lyu B. , Bao H. B. , Jin H. F. , Zhao J. , Xu Y. , and Huang X. , Comparison of Different Serological Methods in Screening Early Gastric Cancer, Zhonghua Nei Ke Za Zhi. (2019) 58, no. 4, 294–300, 10.3760/cma.j.issn.0578-1426.2019.04.011, 30917423. 30917423

[21] Cai Q. , Zhu C. , Yuan Y. , Feng Q. , Feng Y. , Hao Y. , Li J. , Zhang K. , Ye G. , Ye L. , Lv N. , Zhang S. , Liu C. , Li M. , Liu Q. , Li R. , Pan J. , Yang X. , Zhu X. , Li Y. , Lao B. , Ling A. , Chen H. , Li X. , Xu P. , Zhou J. , Liu B. , Du Z. , Du Y. , Li Z. , Gastrointestinal Early Cancer Prevention , Treatment Alliance , and of China (GECA) , Development and Validation of a Prediction Rule for Estimating Gastric Cancer Risk in the Chinese High-Risk Population: A Nationwide Multicentre Study, Gut. (2019) 68, no. 9, 1576–1587, 10.1136/gutjnl-2018-317556, 2-s2.0-85063755755, 30926654. 30926654 PMC6709770

[22] Kang J. M. , Kim N. , Yoo J. Y. , Park Y. S. , Lee D. H. , Kim H. Y. , Lee H. S. , Choe G. , Kim J. S. , Jung H. C. , and Song I. S. , The Role of Serum Pepsinogen and Gastrin Test for the Detection of Gastric Cancer in Korea, Helicobacter. (2008) 13, no. 2, 146–156. 18321304 10.1111/j.1523-5378.2008.00592.x

[23] Ito M. , Yoshihara M. , Takata S. , Wada Y. , Matsuo T. , Boda T. , Tanaka S. , and Chayama K. , Serum Screening for Detection of High-Risk Group for Early-Stage Diffuse Type Gastric Cancer in Japanese, Journal of Gastroenterology and Hepatology. (2012) 27, no. 3, 598–602. 21883453 10.1111/j.1440-1746.2011.06893.x

